# The later stages of viral infection: An undiscovered country of host dependency factors

**DOI:** 10.1371/journal.ppat.1008777

**Published:** 2020-08-25

**Authors:** Cason R. King, Andrew Mehle

**Affiliations:** Medical Microbiology and Immunology, University of Wisconsin–Madison, Madison, Wisconsin, United States of America; University of Kentucky, UNITED STATES

## Virus and host: An unhealthy relationship

With their limited size and coding capacity, all viruses must exploit their host cells for the machinery and raw material they require for replication. At the same time, infected cells deploy an armada of antiviral effector proteins the virus must evade or counteract. This back-and-forth assault, commonly referred to as an “arms race,” impacts viral replication, tropism, and disease severity [1]. How viruses engage with their intracellular environments at the molecular level forms an overlapping research landscape for both virology and cell biology.

Host factors are proteins encoded by cellular genes that affect an intruding virus. Those that ablate viral replication or spread are antiviral factors, while those that enable or enhance the infection perform pro-viral roles and are host dependency factors (HDFs). All of these participate in the battle between pathogen and host and are often under strong evolutionary selection. The identities and functions of these proteins can be as unique and diverse as viruses themselves. It is unsurprising that despite development of powerful systems-level methodologies, we still possess an incomplete knowledge of the virus–host interface for even the best-studied pathogens.

## What we do and do not know about HDFs

Antiviral factors involved in intrinsic or innate immune signaling as well as host restriction have been studied in great molecular depth for many viruses that cause human disease. These are perhaps best exemplified by the interferon-stimulated genes (ISGs), which number in the dozens, if one considers only core or ancestral ISGs, or up to hundreds or even thousands with more generous definitions, depending on the cell and tissue type [2]. While we may have comprehensive lists of antiviral factors, their specific mechanisms of action are not fully described and may even vary depending on the virus or the specific host.

Individual HDFs have been discovered and characterized for many viral systems [3–6]. Despite impressive advances in systems-level experimental approaches, there exists a large knowledge gap on the true breadth of HDFs for both lytic and latent viruses. The elucidated functions for many HDFs discovered to date often occur in the early phases of infection, e.g. during attachment, entry, or early trafficking events. There have also been important studies of HDFs involved in latency and reactivation of long-term infection (see, for example, [7,8]). We focus here on lytic viral replication and the comparatively underexplored HDFs specifically required at later stages of infection. This raises our main question: What is the full repertoire of HDFs usurped by viruses to enhance viral processes in the middle-to-late phases of infection, including viral transcription, splicing, nucleic acid transport, translation, assembly, egress, and others?

## How do we find HDFs?

The term “undiscovered country” in the title makes passing reference to Hamlet’s most famous soliloquy. The bard used it to illustrate our ignorance of and hesitation to explore the afterlife. We find these themes on exploration analogous to our knowledge gap of HDFs regulating later stages of viral infections. The “undiscovered country” of HDFs during later stages of replication exists not due to fear but the technical and biological limits of our methods.

Earlier discovery-based methods, like random gene perturbation or ectopic overexpression with complementary DNA (cDNA) libraries, have given way to more targeted and high-throughput techniques. While many techniques exist for studying both genetic and physical interactions between a virus and its host [6], here, we focus briefly on genetic screens and selections. Genetic screens and selections are a powerful means of identifying important pro- and antiviral host factors in a high-throughput manner [9]. By linking individual perturbation of genes across a genome to some measurable phenotypic output, such as viral gene expression, these experiments quickly narrow the field to identify “hits” for detailed study. However, despite robustness, screens and selections are subject to inherent technical limitations. Those relying on loss-of-function approaches like RNA-interference (RNAi), gene-knockouts, and haploid screens often fail to detect the factors essential for cell viability and factors that still function in very small quantities and have difficulty differentiating between factors with redundant functions [10,11]. RNAi-based screens, in particular, can suffer from high off-target activity, false-positive and -negative results, and incomplete penetrance [11].

Programmable nucleases, like CRISPR/Cas9 systems, have helped overcome many limitations for both gain- and loss-of-function screens [12,13]. CRISPR/Cas9 systems have been engineered for high activity and specificity, enabling complete knockout of target genes. In addition, catalytically inactive Cas9 has been exploited to recruit factors to target sites in the genome. This approach is frequently used to modify transcription of a target gene by recruiting transcriptional activators or inhibitors (CRISPR activation [CRISPRa] or CRISPR inhibition [CRISPRi], respectively). While RNAi, knockout approaches, and CRISPRi ablate gene expression, a major advantage of CRISPRa is its up-regulation of targeted host genes and the ability to perform new types of overexpression and gain-of-function screens [14].

Almost all of these highlighted approaches rely on genetic perturbation prior to infection and screening. Hits from these approaches frequently display a strong bias towards factors affecting the very earliest stages of infection, including attachment and entry. This is in part due to the key role of these events during infection but also a technical artifact that many screens use reporter or single-cycle version of the virus of interest that do not fully capture the full replicative cycle. Despite this limitation, these approaches have provided tremendous insights into entry factors or receptors for many viruses, such as Epstein–Barr virus [15], human immunodeficiency virus (HIV) [16], adeno-associated virus (AAV) [17] influenza A virus (IAV) [5,18], noroviruses [19], flaviviruses [20,21], enteroviruses [22], alphaviruses [23], and hemorrhagic fever viruses [24,25]. Nonetheless, new approaches are needed to better characterize the later stages of their replication cycles.

## How is innovation widening the lens of discovery?

### Camouflaged viruses

**Fig 1:**
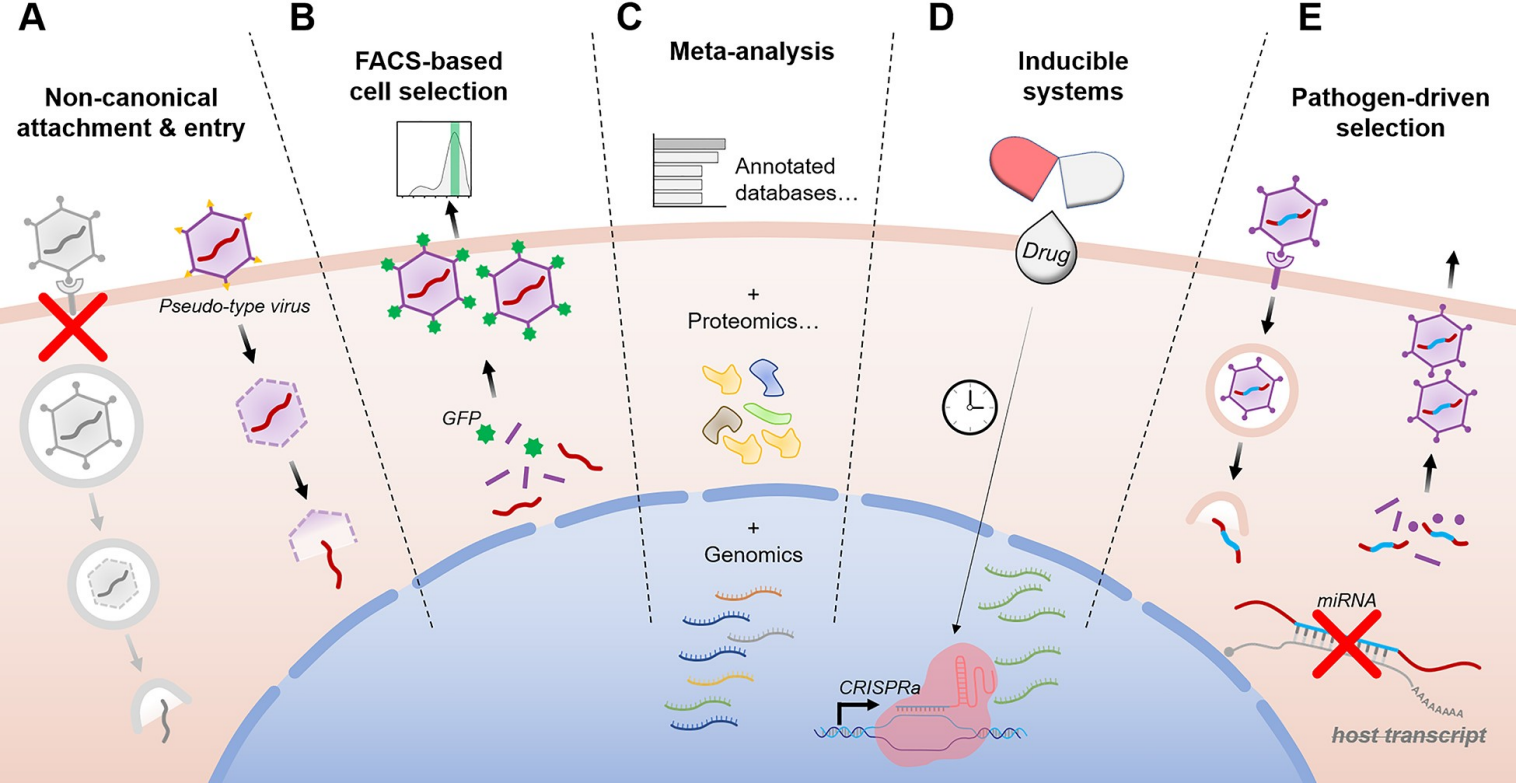
Screening strategies for studying later stages of viral infections and avoiding rediscoveries. **(A)** Pseudotyped viruses bypass host factors affecting canonical attachment and entry events. **(B)** FACS-based selection of reporter genes (such as GFP shown here) or endogenous viral protein expression studies noncytolytic pathogens or specific timepoints postinfection and does not rely on cell survival as a phenotype. **(C)** Meta-analysis increases statistical power and reprioritizes hits by combining data from multiple studies or different systems-level techniques. **(D)** Regulatable screening platforms manipulate host genes at defined time points to query discrete stages of the viral life cycle, as exemplified by the *dox-*inducible CRISPRa system shown here. Light-inducible, cleavage-activated, and complementation-based regulation strategies also exist. **(E)** Virus-driven selections are possible when the virus itself alters endogenous host genes postinfection (for example, by using a virally encoded miRNA to repress a target transcript). The effect of these alterations will alter the replication fitness of the encoding virus, changing its abundance over the course of replication. CRISPRa, CRISPR activation; FACS, fluorescence-activated cell sorting; GFP, green fluorescent protein; miRNA, microRNA.

Numerous advances continue to emerge to help navigate around technical and biological limitations. One strategy to limit early stage biases and rediscovery of known receptors and entry factors is to bypass canonical attachment and entry pathways. For enveloped viruses such as HIV or IAV, this can be achieved by replacing native envelope proteins with those from a different virus, like murine leukemia virus or vesicular stomatitis virus, to generate pseudotyped particles [26,27]. While this does not completely eliminate the rediscovery of entry factors, this bias can be minimized by pseudotyping with viral glycoproteins that use multiple or redundant entry pathways and are thus less likely to emerge from screens using single-gene perturbations. Pseudotyping has seen widespread, successful use in both screening and validation experiments (Fig 1A).

### Alternative selection methods

Genetic investigations of HDFs in virally-infected cells have mostly comprised survival-based selections. However, other experimental strategies and phenotypic endpoints can be chosen. For instance, detection of viral protein expression at a specific time postinfection using fluorescence-activated cell sorting (FACS) or microscopy can be used as a readout for viral replication (Fig 1B). Cells binned according to differences of viral protein production can be analyzed to identify the host factors responsible. This has already been used to successfully identify HDFs for a range of viruses, including HIV, AAV, and IAV [16,17,27]. Sorting avoids reliance on cell death as a binary phenotypic readout, enabling easier screening of noncytolytic pathogens as well as more subtle changes along a spectrum of viral gene expression. With careful experimental design and selection of timepoints, cell populations could be examined to selectively identify changes in early or late gene expression. This fine-grained approach has the potential to reveal positively and negatively enriched host genes within discrete time windows of the viral replication cycle.

### Meta-analyses

An emergent benefit of open science is the ability to aggregate large-scale results from different experiments and techniques that addressed similar questions (Fig 1C). Meta-analysis can discover unknown HDFs in existing datasets and reprioritize them for experimental validation [28,29]. For instance, Li and colleagues combined findings from multiple screens, protein–protein interaction databases, and annotated pathways to reveal a host methyltransferase that was involved in IAV cap-snatching—a decidedly “middle” stage of viral replication [27]. Another study combined a CRISPR screen with proteomic analysis to prioritize a host factor required for norovirus protein translation [30]. And newer techniques, such as global characterization of physical interactions between viral RNA and host proteins, were combined with a genome-wide CRISPR screen to define new host factors during middle-to-late stages of flavivirus infection [31]. The overall findings in each case were still subject to the biological limits imposed by a loss-of-function knockout screen; however, these examples demonstrate how meta-analyses can reveal already large datasets to be even richer than initially observed.

### Timing is everything

When an HDF is expressed and how much is made can drastically change its biological effect. This information is lost where the genetic manipulation is static, like a gene knockout or constitutive CRISPRa cell line. Experimentally manipulating HDF timing and abundance enables probing of additional aspects of viral replication, while, at the same time, minimizing the bias towards early stages of infection. A suite of tools has been developed for inducible and tunable alterations to target gene expression on a genome-wide scale at times chosen by the researcher (Fig 1D). For Cas9-based systems, drug-, temperature-, light-, and molecular switch-based versions have been used to precisely control expression and timing [32]. Importantly, transcriptional changes occur on the timescale of even the fastest viral infections [33,34], allowing regulated CRISPRa and CRISPRi screens to focus on HDFs affecting specific postentry events.

### Make the virus do the “heavy lifting”

An alternative approach is to fool the virus into revealing its own Achilles’ heel(s). Through clever molecular virology, the virus under study is engineered to perturb targeted host genes itself (Fig 1E). In contrast to the more straightforward loss-of-function experiments, this strategy has been used in gain-of-function selections. Pools of viruses targeting unique genes are competed in a fitness-based screen. The relative abundance of a given virus after the screen reflects its fitness and the impact of the targeted host factor on replication. A notable example of this strategy involved engineering replication-competent alphaviruses to encode artificial microRNAs capable of silencing endogenous target genes in mice [35]. Passaging experiments with these viruses pressured them to reveal antiviral effectors of alphavirus replication; viruses encoding miRNAs that suppressed antiviral effectors gained a replicative advantage and quickly dominated the population. This was similarly applied to IAV where competition-style experiments “tricked” IAV into revealing MDA5 as a potent antiviral factor [36].

A more recent study utilized HIV capable of packaging single-guide RNAs (sgRNAs) into progeny virions [26]. Recombinant lentivirus genomes containing sgRNAs were first used to generate pooled CRISPR knockout cells. Cells were then infected with bona fide HIV, which budded new HIV particles containing the sgRNA-encoding genomes expressed from the knockout vector resident in the cell. The amount of each sgRNA present in the pool of released virions provided a quantitative measure of the pro- or antiviral activity of the targeted gene. Attachment and entry factors emerged as top HDFs; nonetheless, the approach generated significant results in only a single round of viral replication, was readily adapted to multiple HIV strains, and could potentially be applied to other viral systems. Moreover, if the approach can be adapted such that released sgRNA-encoding genomes are fully replication-competent, multiple rounds of reinfection and selection can further enrich top hits.

## Concluding remarks

Like their antiviral counterparts, HDFs can dictate the success or failure of infections. This can be based on their expression levels, allelic profiles, and splicing patterns. Importantly, the evolution of eukaryotic genes is significantly slower than that of a virus [37]. Therefore, undiscovered HDFs may offer more stable targets for the development of new antiviral therapeutics. This strategy has been successfully implemented in the design of antiretroviral drugs targeting the host coreceptor CCR5 [38]. Additionally, targeting key HDFs can reveal broadly-acting compounds like GSK983 that are effective against diverse pathogens, providing an alternative to the “one bug, one drug” approach [39,40]. Being able to comprehensively survey the entire spectrum of HDFs affecting all stages of viral replication will further enable host-directed therapies. Continued innovation in approaches to identify HDFs will begin to fill gaps in our knowledge about later stages of viral infection and provide new solutions to previously intractable problems.
